# Inactivated tick-borne encephalitis vaccine elicits several overlapping waves of T cell response

**DOI:** 10.3389/fimmu.2022.970285

**Published:** 2022-08-24

**Authors:** Anastasiia L. Sycheva, Ekaterina A. Komech, Mikhail V. Pogorelyy, Anastasia A. Minervina, Shamil Z. Urazbakhtin, Maria A. Salnikova, Mikhail F. Vorovitch, Eugene P. Kopantzev, Ivan V. Zvyagin, Alexander Y. Komkov, Ilgar Z. Mamedov, Yuri B. Lebedev

**Affiliations:** ^1^ Department of Genomics of Adaptive Immunity, Shemyakin-Ovchinnikov Institute of Bioorganic Chemistry RAS, Moscow, Russia; ^2^ Department of Molecular Technologies, Institute of Translational Medicine, Pirogov Russian National Research Medical University, Moscow, Russia; ^3^ Department of Immunology, St. Jude Children’s Research Hospital, Memphis, TN, United States; ^4^ Computational Systems Biochemistry Research Group, Max Planck Institute of Biochemistry, Martinsried, Germany; ^5^ Department of Immunology, Faculty of Biology, Lomonosov Moscow State University, Moscow, Russia; ^6^ Laboratory of Tick-Borne Encephalitis and Other Encephalitis, Chumakov Federal Scientific Center for Research and Development of Immune-and-Biological Products of RAS (FSASI “Chumakov FSC R&D IBP RAS”), Moscow, Russia; ^7^ Department of Organization and Technology of Production of Immune-and-Biological Products, Institute for Translational Medicine and Biotechnology, Sechenov First Moscow State Medical University, Moscow, Russia; ^8^ Department of Genomics and Postgenomic Technologies, Shemyakin-Ovchinnikov Institute of Bioorganic Chemistry RAS, Moscow, Russia; ^9^ Laboratory of Cytogenetics and Molecular Genetics, Dmitry Rogachev National Medical and Research Centre of Paediatric Haematology, Oncology and Immunology, Moscow, Russia

**Keywords:** TCR repertoire, T cell immune response, clonal expansion, immunological memory, TCR motif, TBE vaccination, tick-borne encephalitis

## Abstract

The development and implementation of vaccines have been growing exponentially, remaining one of the major successes of healthcare over the last century. Nowadays, active regular immunizations prevent epidemics of many viral diseases, including tick-borne encephalitis (TBE). Along with the generation of virus-specific antibodies, a highly effective vaccine should induce T cell responses providing long-term immune defense. In this study, we performed longitudinal high-throughput T cell receptor (TCR) sequencing to characterize changes in individual T cell repertoires of 11 donors immunized with an inactivated TBE vaccine. After two-step immunization, we found significant clonal expansion of both CD4^+^ and CD8^+^ T cells, ranging from 302 to 1706 vaccine-associated TCRβ clonotypes in different donors. We detected several waves of T cell clonal expansion generated by distinct groups of vaccine-responding clones. Both CD4^+^ and CD8^+^ vaccine-responding T cell clones formed 17 motifs in TCRβ sequences shared by donors with identical HLA alleles. Our results indicate that TBE vaccination leads to a robust T cell response due to the production of a variety of T cell clones with a memory phenotype, which recognize a large set of epitopes.

## 1 Introduction

Vaccination is one of the greatest achievements of healthcare, which has contributed to a significant increase in life expectancy over the past century (1). However, many infections still pose a threat, and we regularly face new challenges, one of which is the recently emerged novel coronavirus disease (COVID-19). Significant resources have been invested to combat this infection and all available technologies for vaccine development have been used, including a classical approach of creating inactivated vaccines, which have a good safety and tolerance profile, as well as mild storage and transport requirements. For each new vaccine, including an inactivated one, an immunogenicity test is required, in which the level of specific antibodies is the major evaluation parameter (2). However, complete elimination of viral pathogens is possible only with the involvement of T cell response (3), which should be considered in the process of developing inactivated vaccines. Furthermore, T cells play a role in the differentiation of long-lived plasma cells and generation of high-affinity antibodies (4) and can recognize many conserved regions of viral polymerases (5) and other proteins essential for the virus life cycle but inaccessible to antibodies.

Conventional T cells with αβ T cell receptors (TCRs) play an important role in infection control, pathogen elimination, and establishment of long-lived immunological memory. The high diversity of the TCR repertoire is provided by V(D)J-recombination mechanisms, which theoretically allow generation of up to 2 × 10^19^ of unique αβTCRs (6). The reorganization of the TCR repertoire upon infection or vaccination reflects changes in the abundance and phenotypic properties of responding clonotypes. The development of high-throughput sequencing technologies has significantly improved the quality of TCR repertoire profiling, making it possible to detect any minor changes in T cell-mediated immunity. Studies that used deep TCR repertoire profiling and clonotype tracking to assess immune response to vaccination have been first performed on live attenuated vaccines (7–10) known to induce strong T cell response, and later, on other vaccine types (11–15). However, the data on the effect of multi-step vaccination on the human TCR repertoire and immunological T cell memory are still limited.

Vaccination against tick-borne encephalitis (TBE) is an example of a highly effective immunization schedule including administration of multiple doses of the inactivated vaccine (16). Still, regular booster vaccination is required to maintain a high level of protection. Breakthrough TBE infections are a matter of concern in endemic regions because of a high risk of neurological complications and even death (17). To overcome drawbacks of the current vaccination protocol and develop new generation TBE vaccines, better understanding of the mechanisms underlying T cell response to TBE vaccination is needed. In this study, we performed deep profiling and longitudinal tracking of the TCRβ repertoire after two-step immunization with the inactivated TBE vaccine. We observed hundreds of vaccine-associated CD4^+^ and CD8^+^ T cell clones, with a prevalence of CD4^+^ responding cells. The clonotypes had highly diverse expansion dynamics and could be subdivided into four groups with different roles in immune response and distinct contributions to memory T cell generation. Despite variability in individual immune responses, we observed several common patterns, including clusters of vaccine-associated clonotypes with similar TCRβ amino acid sequences in different donors.

## 2 Materials and methods

### 2.1 Donors and blood sample collection

**Figure 1:**
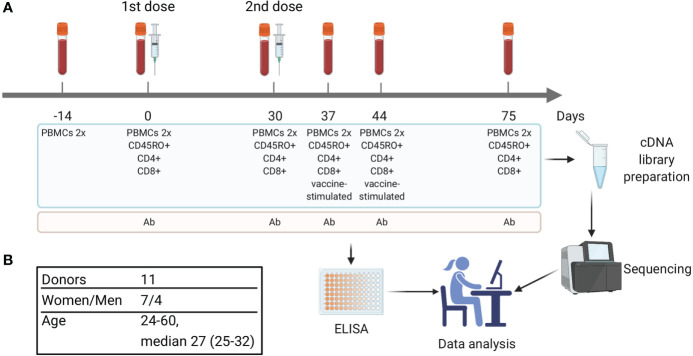
Study design. **(A)** Timeline of vaccination and blood sample collection. At each time point two replicates of bulk PBMCs were collected (PBMCs 2x). Additional aliquots of PBMCs were divided into cell subsets, including CD4^+^, CD8^+^, and CD45RO^+^ (memory T cells). On days 37 and 44 a portion of PBMCs was used for *in vitro* stimulation with inactivated purified TBEV particles followed by isolation of CD137^+^ and IFNγ-producing T cells. Blood serum was used for TBEV-specific IgG antibody level measurement. **(B)** Donors cohort information.

All subjects gave written informed consent in accordance with the Declaration of Helsinki. The study was approved by the Pirogov Russian National Research Medical University local ethics committee. Informed consent was obtained from 11 healthy donors (7 women and 4 men, age 24–60 years [median 27 years, Q1 = 25 – Q3 = 32]). Each donor received two doses of the inactivated TBE vaccine (Tick-E-Vac^®^, 0.5 ml, FSBSI “Chumakov FSC R&D IBP RAS”, Russia) with the interval of 30 days according to the approved immunization protocol (16); immunizations were performed in 2016–2018. Ten donors had no record of previous TBE vaccinations or infections, and one (donor #9) had a TBE vaccination course 10 years prior to the study. Blood samples (9–72 ml) were collected at each time point (**Figure 1**) in a certified diagnostics laboratory and placed into VACUETTE^®^ K3E K3EDTA tubes (Greiner Bio-One, Austria). Additional portions of peripheral blood (from 2.5 to 10 ml) were collected into the VACUETTE^®^ CAT serum separator clot activator tubes (Greiner Bio-One, Austria) for serum isolation according to a standard protocol. HLA alleles for all donors were determined using an in-house cDNA high-throughput sequencing method.

### 2.2 Isolation of PBMCs and T cell subsets

Blood samples from each donor at each time point were divided into 4 aliquots of 4.5 ml for parallel PBMCs preparation and subsequent isolation of bulk, CD4^+^, CD8^+^ and CD45RO^+^ T cell subsets. PBMCs were isolated by Ficoll-Paque (Paneco, Russia) density gradient centrifugation according to a standard protocol. CD4^+^, CD8^+^, and CD45RO^+^ T cell subsets were isolated from PBMCs using the CD4 and CD8 Dynabeads Positive Isolation Kits (Invitrogen, USA) and CD45RO MicroBeads (Miltenyi Biotec, USA), respectively, according to the manufacturers’ protocols. Obtained T cell subsets and bulk PBMCs were lysed using Trizol reagent (Invitrogen, USA) for total RNA isolation. Additional blood samples were collected on days 37 and 44. Corresponding PBMCs were used for *in vitro* stimulation and cell sorting of activated T cell subsets (see below for details).

### 2.3 *In vitro* T cell stimulation and flow cytometry

PBMCs were diluted to 1 × 10^7^ cells/mL in RPMI 1640 GlutaMAX Supplement medium (Gibco, USA) with 5% autologous serum and stimulated with inactivated purified TBEV particles, Sofjin strain (18) (10 μg/mL of E protein determined using VectoTBEV-antigen kit [Vector-Best, Russia]). Stimulated cells were incubated in 24-well plates for 16 or 24 h at 37°C and 5−7% CO_2_. After 16 h, half of the stimulated cells were subjected to the IFNγ Secretion Assay (Miltenyi Biotec, USA) according to the manufacturer’s protocol. After 24 h, the rest of the cells were washed and stained with PE-labeled anti-CD137 antibodies (Miltenyi Biotec, USA, clone REA765). In addition, cells were stained with eFluor450-labeled anti-CD3 antibodies (eBioscience, USA, clone UCHT1). Then, CD3^+^IFNγ^+^ and CD3^+^CD137^+^ cells were collected by fluorescence-activated cell sorting on Sony SH800S Cell Sorter with the Purity Mode allowing for at least 97% of purity (see **Supplementary Figure 1** for the gating strategy and **Supplementary Table 1** for the number of sorted cells) and lysed with Trizol reagent (Invitrogen, USA) immediately followed by total RNA isolation.

### 2.4 ELISA

Serum samples collected on days 0, 30, 37, 44, and 75 (**Figure 1**) were used for detection and quantification of TBEV-specific IgGs with VectoTBEV-IgG kit (Vector-Best, Russia). Each sample was tested twice.

### 2.5 TCRβ library preparation and sequencing

The preparation of TCRβ-chain cDNA libraries for bulk PBMCs, CD4^+^, CD8^+^, and CD45RO^+^ T cells was performed as previously described (19–21). In brief, first-strand cDNA was synthesized from total RNA using SmartScribe revertase (Clontech, USA) and universal primers specific for the TCRβ-chain constant segment. SMART oligonucleotides were used to generate universal 5′-ends and to introduce unique molecular identifiers (UMIs). Two-step PCR was used to amplify cDNA and introduce Illumina adapters at the amplicon ends. PCR products were purified using the Qiagen PCR purification kit (Qiagen, Germany).

TCR cDNA libraries for IFNγ-producing and CD137^+^ T cell subsets were prepared as previously described (22) using SuperScript™ III Reverse Transcriptase (Invitrogen, USA) for cDNA synthesis and Human multiplex TCR kit (MiLaboratories, USA) for cDNA amplification. PCR products were purified using the Qiagen PCR purification kit.

cDNA libraries were sequenced on Illumina HiSeq (2x100nt) and NovaSeq (2x150nt) platforms. The total number of sequencing reads for each sample is shown in **Supplementary Table 1**.

### 2.6 TCR repertoire data analysis

#### 2.6.1 Raw data preprocessing

For TCRβ libraries prepared by the 5′ RACE technology, raw sequencing data files were preprocessed with MiNNN v10.1 (https://github.com/milaboratory/minnn), and sequencing reads were clustered by UMI. Sequences were processed with MiXCR v3.0.13 (23) to extract TCR CDR3 sequences, determine V, D, and J genes, and build clonotypes. Numbers of UMIs and clonotypes after filtering are shown in **Supplementary Table 1**.

#### 2.6.2 Identification of vaccine-associated clonotypes

Statistically significant expanded and contracted clonotypes in TCRβ repertoires of bulk PBMCs were identified with ‘edgeR’ package (24) as previously described (9). In brief, two bulk TCRβ repertoires were used as biological replicates for each time point. Normalization by the TTM method and trended dispersion estimation were performed according to the ‘edgeR’ User’s Guide. To identify significant differences in expanded or contracted clonotypes between two time points, we used an exact test based on the qCML methods and FDR BH-adjusted p-value ≤ 0.01. Clonotypes with log2 fold-change ≥ 5 between two time points and absent in TCRβ repertoires of bulk PBMCs before vaccination (days -14 and 0) were considered as vaccine-associated.

To identify clonotypes that underwent antigen-driven selection, TCRβ repertoires of bulk PBMCs collected on days 30, 37, and 44 were analyzed using the ALICE pipeline in combination with OLGA (25, 26). Clonotypes predicted by ALICE with BH-adjusted p-value < 0.001 and not present in the TCRβ repertoires before vaccination were also considered vaccine-associated.

#### 2.6.3 Comparison of TCRβ amino acid sequences

We calculated the Hamming distance for pairs of CDR3 amino acid sequences and marked clusters of similar clonotypes (identical V and J segments with one or no mismatch in CDR3 amino acid sequences). Before comparison of CDR3 amino acid sequences of different donors, we checked if any of the vaccine-associated clonotypes with given V and J segments and at most 1 amino acid mismatch could be found in the VDJdb database (27) by using the VDJmatch v1.3.1 (28). The search was conducted for the records of clonotypes with similar CDR3 amino acid sequences (one or no substitutions), identical V and J segments and CDR3 length. We only considered TCR clonotypes for which HLA allele restriction in VDJdb matched one of the alleles of the donors bearing this TCR. All matched clonotypes were excluded from further analysis.

Random subsets of clonotypes were generated by sampling from replicates of bulk TCRβ repertoires on days 30, 37, and 44. We repeated the random sampling 100 times for each time point. The mean number of pairs of similar clonotypes in the random subset was compared with the number of pairs of similar clonotypes in the vaccine-associated subset.

## 3 Results

### 3.1 Study design

Eleven healthy donors were immunized with the inactivated TBE vaccine (Tick-E-Vac^®^, FSBSI “Chumakov FSC R&D IBP RAS”) twice with a 30-day interval according to the approved immunization protocol. Ten donors had no record of previous TBE vaccinations or infections, whereas one (donor #9) received a TBE vaccination course 10 years prior to the study. Blood samples were collected before the 1st (days -14 and 0) and 2nd (day 30) doses and on days 37, 44, and 75 (**Figure 1**) and used to isolate peripheral blood mononuclear cells (PBMCs) in two biological replicates. To detect T cell clonal expansion we chose two time points (days 37 and 44) because T cell response has been demonstrated 1 and 2 weeks after immunization with another inactivated TBE vaccine in an earlier study (29). Aliquots of blood samples from days 0, 30, 37, 44, and 75 were also used to isolate CD4^+^, CD8^+^, and CD45RO^+^ (memory) T cell subpopulations. PBMCs obtained from two post-vaccination samples (days 37 and 44) were used for *in vitro* stimulation with inactivated purified TBE virus (TBEV) particles with subsequent isolation of CD137^+^ and IFNγ^+^ T cell subsets. All obtained cells were used for TCR cDNA library preparation and high-throughput Illumina sequencing. TCRβ repertoires were reconstructed using computational methods with PCR/sequencing error correction. The level of TBEV-specific IgG antibodies was measured in serum samples collected on days 0, 30, 37, 44, and 75 (**Figure 1**).

### 3.2 Two-step immunization with the inactivated TBE vaccine induces strong immune response and generation of memory T cells

**Figure 2:**
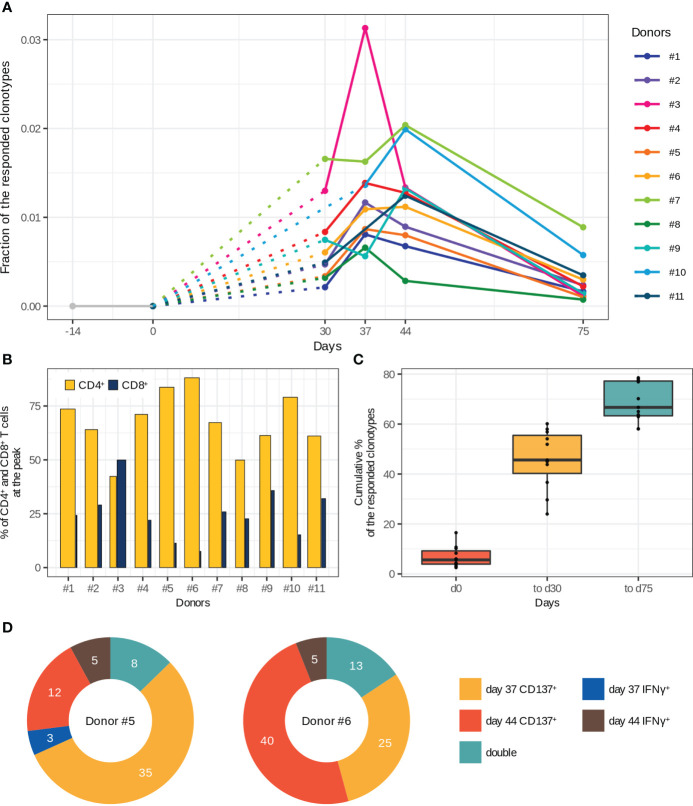
Characteristics of T cell immune response to two-step immunization with the inactivated TBE vaccine. **(A)** Dynamics of responded clonotype fractions in the bulk TCRβ repertoire calculated as the sum of clonotype frequencies (average of two replicates at each time point). **(B)** Percentage of CD4^+^ and CD8^+^ T cells from the fraction of responded T cells at the peak of expansion. The bar width corresponds to the CD4^+^ or CD8^+^ T cell clones. **(C)** Cumulative percentage of responded clonotypes found in TCRβ repertoires of memory (CD45RO^+^) T cell subsets among the responded clonotypes. **(D)** Vaccine-associated clonotypes found in TCRβ repertoires of CD137^+^ and IFNγ-producing T cells obtained after *in vitro* stimulation with inactivated purified TBEV particles on days 37 and 44 for donors #5 and #6. The numbers of detected clonotypes are indicated. For more details about the detection of “double” clonotypes in the activated T cell subsets see **Supplementary Figure 5**.

To examine T cell clonal expansion in response to vaccination, we compared TCRβ repertoires of bulk PBMCs from different time points using an approach we previously described for yellow fever vaccination (9). The approach is based on the active dynamics of immune response and is designed to determine T cell clones that were absent before vaccination and expand after. To take into account sampling noise, we assessed variability in biological replicates (**Supplementary Figures 2A, B**) using the ‘edgeR’ package (24). We only considered clonotypes that were expanded at least 32-fold (**Supplementary Figure 2C**). As a result, we found from 302 to 1706 vaccine-associated clonotypes, which were absent in TCRβ repertoires before vaccination (days -14 and 0) and which constituted up to 3.1% of an individual TCRβ repertoire at the peak of expansion, indicating strong T cell response to the vaccine. The vaccinated donors demonstrated diverse dynamics of T cell response with the highest abundance of the responded clonotypes on day 37 (donors #1–5, 8) or day 44 (donors #6, 7, 9–11) (**Figure 2A**). For two donors (#7 and #9), we also observed a small peak on day 30. At the same time, the dynamics of TBEV-specific IgG production was uniform for almost all donors, with a peak on day 44 (**Supplementary Figure 3**).

Next, we determined CD4^+^ and CD8^+^ phenotypes for the vaccine-associated clonotypes by matching TCRβ nucleotide sequences of bulk repertoires to those of CD4^+^ and CD8^+^ T cell repertoires. Both CD4^+^ and CD8^+^ subsets of the responded clonotypes had similar dynamics except for those in donors #6, #7, and #9, in which CD8^+^ T cell clones demonstrated two-peak dynamics, whereas CD4^+^ T cell clones had only one peak (**Supplementary Figure 4**). The CD4^+^/CD8^+^ ratio of vaccine-associated T cell clones was significantly biased towards CD4^+^ (both in the clone and cell proportions at the peak of T cell response) for all donors except one (**Figure 2B**). Nevertheless, the CD8^+^ T cell response was strong enough to be detected with our 32-fold expansion threshold, and CD8^+^ T cell clones constituted up to 0.53% of all T cells at the peak of expansion. For donor #3, we observed a comparable expansion in both subsets with a subtle prevalence of CD8^+^ T cells (**Supplementary Figure 4**).

The main purpose of any vaccination is to generate immunological memory for long-term protection against subsequent infections. Therefore, to examine whether the vaccine-associated T cell clones developed a memory phenotype we sequenced CD45RO^+^ (memory) T cell subset. The results indicated that 58–79% of all responded clonotypes were detected at least once in the TCRβ repertoires of memory T cells. We also observed an enrichment of the memory T cell compartment with new clonotypes after the 2nd dose (**Figure 2C**). It should be noted that a small number of the responded clonotypes already had the memory phenotype on day 0. Importantly, up to 70% of the responded clonotypes could be detected in the TCRβ repertoires of memory T cells 1.5 months after the 2nd dose (day 75).

To validate whether the expanded clonotypes indeed recognize antigens from TBEV, we evaluated the TCRβ repertoire of T cells expressing CD137^+^ or producing IFNγ after *in vitro* stimulation of the same donor’s PBMCs with inactivated purified TBEV particles on days 37 and 44 (donors #5 and #6). About 10% of the vaccine-associated clonotypes were found among the TCRβ sequences of *in vitro* activated T cells (**Figure 2D**), indirectly confirming the recruitment of these clones in response to vaccination. Moreover, 8 and 13 responded clonotypes (for donors #5 and #6, respectively) were detected in two repertoires of *in vitro* activated T cells (“double” in **Figure 2D** and **Supplementary Figure 5**). There were no differences between the “activated” and other vaccine-associated clonotypes in V and J segment usage, CDR3 length distribution, and the presence in CD4^+^ or CD8^+^ T cell subsets. However, we observed higher abundance of “activated” clonotypes in the TCRβ repertoire of bulk PBMCs collected on the same day as the activated T cell subset (two-tailed Wilcoxon signed-rank test, Benjamini Hochberg adjusted p-value < 0.0000308). The CD4^+^/CD8^+^ ratio in these clonotypes also indicated the predominance of CD4^+^ T cell clones, even for clonotypes found in the repertoire of IFNγ-producing T cells. In addition, 77.8% and 85.5% of the activated T cell clones were detected in the TCRβ repertoires of memory T cells.

Taken together, these results suggest that the identified vaccine-associated T cell clones were involved in the immune response to the TBE vaccine and induced immunological memory.

### 3.3 Dynamics of the vaccine-associated T cell clones shows four waves of immune response

**Figure 3:**
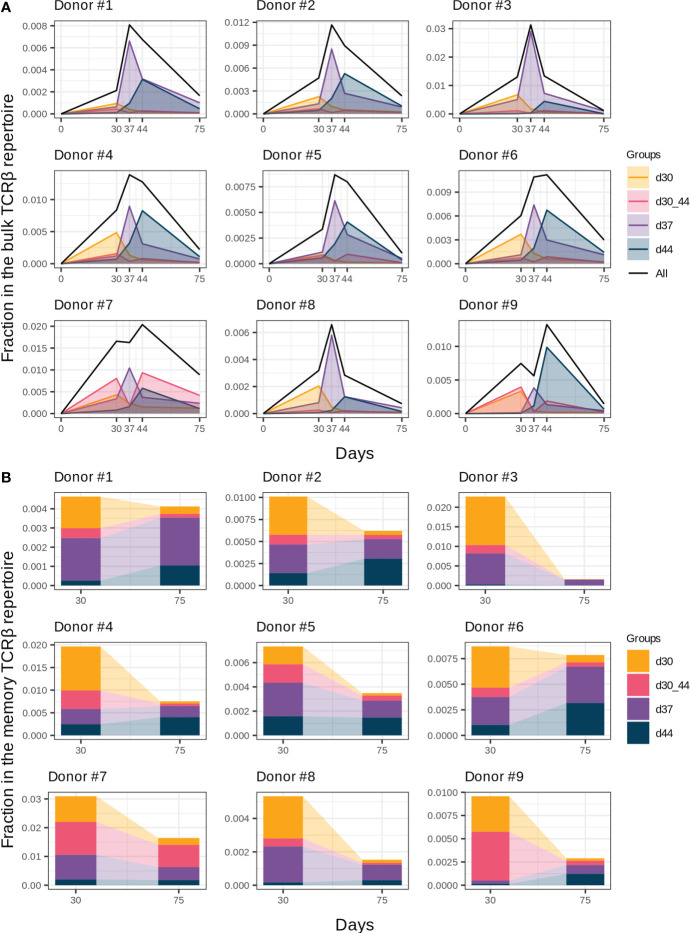
Groups of vaccine-associated clonotypes with different dynamics. **(A)** Dynamics of the responded clonotype fractions in the bulk TCRβ repertoire (**Figure 2A**) formed by clonotypes of groups “d30”, “d37”, “d44”, and “d30_44”. **(B)** Fractions of clonotype groups in memory T cells on days 30 and 75. Donors #10 and #11 were excluded from analysis because of incomplete sample collection.

The general dynamics of the vaccine-associated clonotypes includes several waves of clonal expansion with diverse magnitudes. According to the timing of expansion peaks, we divided all vaccine-associated clonotypes into four groups: d30, d30_44, d37, and d44 (**Figure 3A**). Among them, groups “d37” and “d44” with peaks on days 37 and 44 (i.e., 1 and 2 weeks after the 2nd immunization, respectively) were the most abundant.

In the memory T cell subset on day 30, we detected 40.7 ± 12% and 26.6 ± 15.3% clonotypes of groups “d37” and “d44”, respectively, which makes them the most plausible candidates for reactivated after the 2nd immunization T cell clones. Group “d37” had higher abundance in the memory T cell subsets on day 30 in comparison with group “d44” (**Figure 3B**, paired two-tailed Wilcoxon signed-rank test, p = 0.003906), which could result in a higher proliferating rate after revaccination. We found no statistically significant differences in the CD4^+^/CD8^+^ T cell clone ratio between groups “d37” and “d44” for all donors except #8, for whom the proportion of CD4^+^ T cell clones was significantly lower in group “d44” (G-test, p = 0.0017).

An interesting case was group “d30_44”, which peaked on day 30, contracted one week after (day 37), and expanded once again on day 44. For most donors, it was a minor group of clonotypes (**Figure 3A**); however, for donors #7 and #9, it significantly contributed to the general dynamics of vaccine-associated clonotypes, resulting in “two-peak” expansion with predominance on day 44 (**Figure 2A**). In contrast, clonotypes of group “d30” peaked before the 2nd dose and contracted thereafter despite the 2nd immunization and presence of 70% of its clonotypes in the memory Т cell subset on day 30.

To determine the contribution of the described groups to immunological memory generation, we analyzed clonotype abundance of each group in memory T cell subsets on day 30 (1 month after the 1st dose) and day 75 (1.5 months after the 2nd dose). Despite the extension of the memory compartment with new clonotypes after the 2nd dose (**Figure 2C**), the total fraction of vaccine-associated clonotypes in the memory T cell subset decreased from day 30 to day 75, but still accounted for 0.41% (median, Q1 = 0.29 – Q3 = 0.75) of memory T cells on day 75 (**Figure 3B**). The proportion of the clonal groups in this fraction differed between days 30 and 75, and groups “d37” and “d44” became predominant after restimulation in all donors (except for donor #7 with group “d30_44” prevalence). Thus, it can be assumed that the clonotypes from groups “d37” and “d44” could make the greatest contribution to the establishment of long-term immunity.

### 3.4 Clonotypes with similar TCRβ amino acid sequences have different dynamics

**Figure 4:**
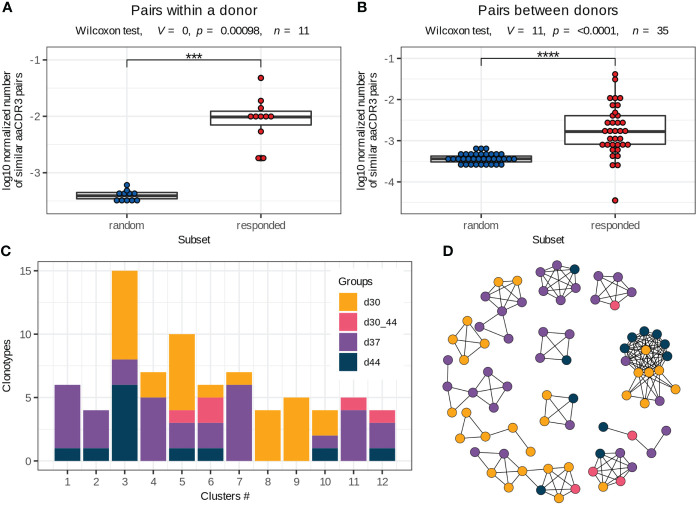
Vaccine-associated clonotypes with similar TCRβ amino acid sequences. **(A, B)** Pairs of similar TCRβ amino acid sequences (identical V and J segments and CDR3 length, with one or no mismatches in CDR3 amino acid sequences) for the responded and random clonotypes within a donor **(A)** and between two donors **(B)** normalized by the number of possible pairs in each subset. Paired two-tailed Wilcoxon signed-rank test was used (***p < 0.001, ****p < 0.0001). **(C)** Group distribution in the clusters of responded clonotypes with similar TCRβ amino acid sequences (example for donor #1). Only 2 of 12 clusters consist of clonotypes of the same group. **(D)** Sequence similarity network for the responded clonotypes of donor #1. Each vertex corresponds to a unique TCRβ clonotype, and edges connect clonotypes with one or no mismatches in CDR3 amino acid sequences and identical V and J segments. Only clusters with 4 or more clonotypes are shown. The color scheme is the same as in **(C)**.

Antigen exposure drives activation and proliferation of clonotypes with identical or similar antigen specificity, which is determined by TCR amino acid sequence (30, 31). In this study, we assessed the similarity of TCRβ by pairwise comparison of CDR3 amino acid sequences in the responded clonotypes with the same V and J segments and CDR3 length. We detected up to 142 clonotypes (median 27, Q1 = 23.5 – Q3 = 38) with one or no amino acid mismatches (the Hamming distance ≤ 1) within the responded clonotypes of the same donor. The vaccine-associated clonotypes formed significantly more pairs with similar TCRs than a random subset of clonotypes with the same VJ-combinations and CDR3 length (**Figure 4A**), suggesting convergent selection of the responded clonotypes.

To extend pools of vaccine-associated clonotypes, we used the ALICE software that detects clonotypes with a higher number of similar TCR amino acid sequences than is expected by the VDJ-recombination model (i.e., in absence of convergent selection) (25). The ALICE was applied to the repertoires of bulk PBMCs, collected on days 30, 37 and 44. Implementation of this approach allowed us to identify about 152 (median, Q1 = 92 – Q3 = 231) additional vaccine-associated clonotypes for each donor. We grouped all responded clonotypes with identical V and J segments and CDR3 length and similar (one or no mismatches) CDR3 sequences within each donor into about 35 clusters (median, Q1 = 28.5 – Q3 = 39.5) (**Supplementary Figure 6**), many of which contained both highly expanded (detected with the ‘edgeR’ package) and slightly expanded (detected with ALICE) clonotypes. Then, we examined the expansion dynamics for clusters consisting of ≥ 4 clonotypes. Only 22% (median, Q1 = 13 – Q3 = 35) of clusters included clonotypes with identical dynamics (**Figures 4C, D**), indicating that the vaccine-associated clonotypes with the same or very similar TCRβ sequences could have distinct functions during immune response.

### 3.5 Responded clonotypes from donors with shared HLA alleles have similar TCRβ amino acid sequences

Donors with shared HLA alleles (**Supplementary Figure 7**, **Supplementary Table 2**) were immunized with the same vaccine, suggesting that they should have identical or very similar peptide-MHC complexes and, consequently, similar TCRs of T cell clones involved in immune response. To filter out shared clonotypes specific to other widespread pathogens, we screened the vaccine-associated clonotypes against the VDJdb database (27). All matched clonotypes were excluded from further analysis.

**Figure 5:**
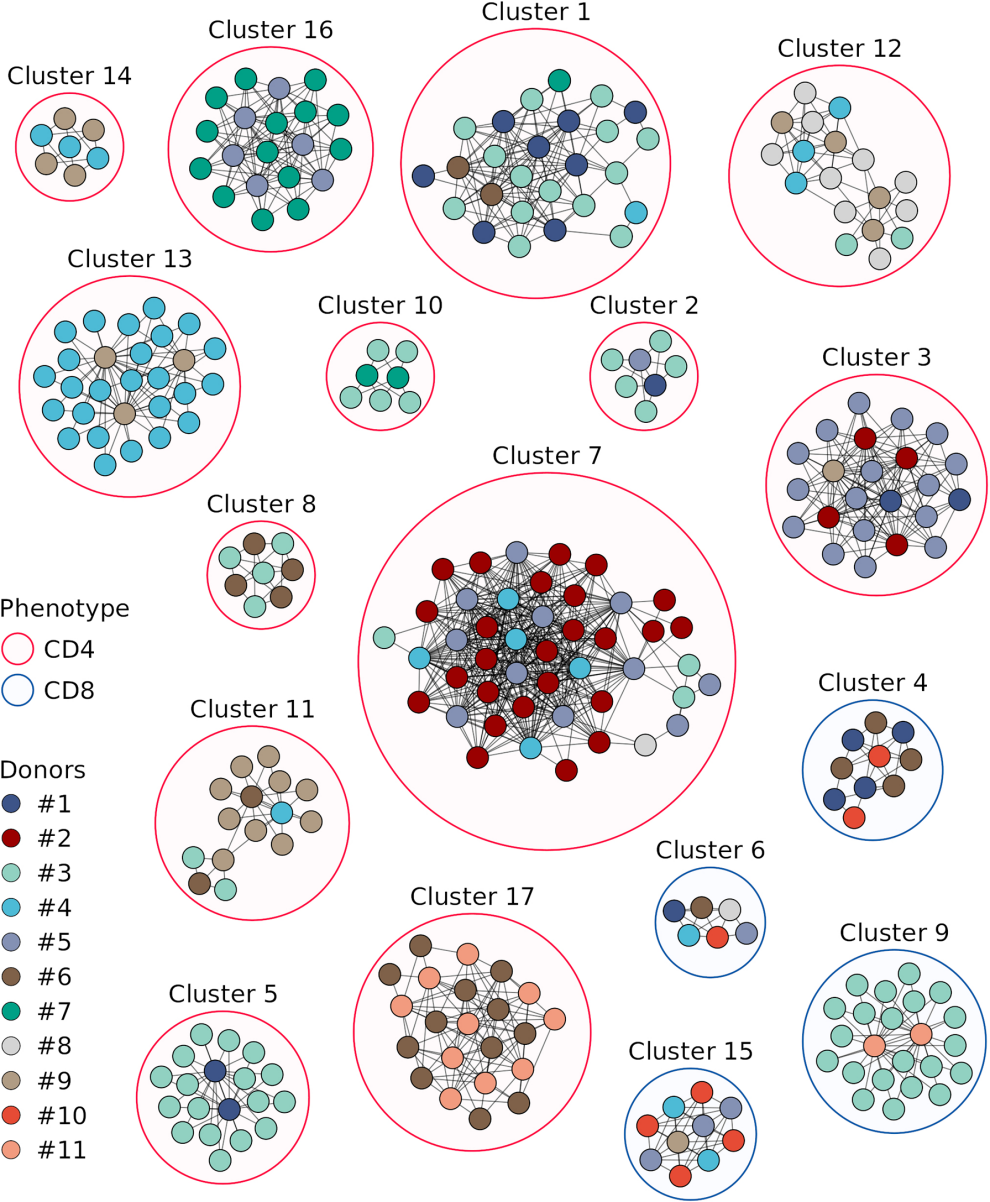
Sequence similarity networks for the responded clonotypes from different donors. Each vertex corresponds to a unique TCRβ clonotype. Edges connect clonotypes with one or no mismatches in CDR3 amino acid sequences and identical V and J segments; only edges between clonotypes of different donors are shown. Color of the area around clusters indicates CD4^+^ (red) or CD8^+^ (blue) phenotypes of T cell clones in the cluster. Only clonotypes with similarity to at least 2 clonotypes of other donors and clusters with 6 or more clonotypes are displayed.

We next compared TCRβ amino acid sequences of the vaccine-associated clonotypes from different donors and revealed 17 clusters consisting of 6 or more clonotypes, which were similar to at least 2 clonotypes from other donors (identical V and J segments and CDR3 length, one mismatch in the CDR3 amino acid sequence allowed) (**Figure 5**, **Supplementary Table 3**). Among the 17 clusters, 9 included vaccine-associated clonotypes from the repertoires of more than 2 donors. For each clonotype pair, we tested whether they could have been detected by chance because of similarity between donor TCRβ repertoires and found that randomly sampled clonotypes had significantly less similar pairs (**Figure 4B**).

**Figure 6:**
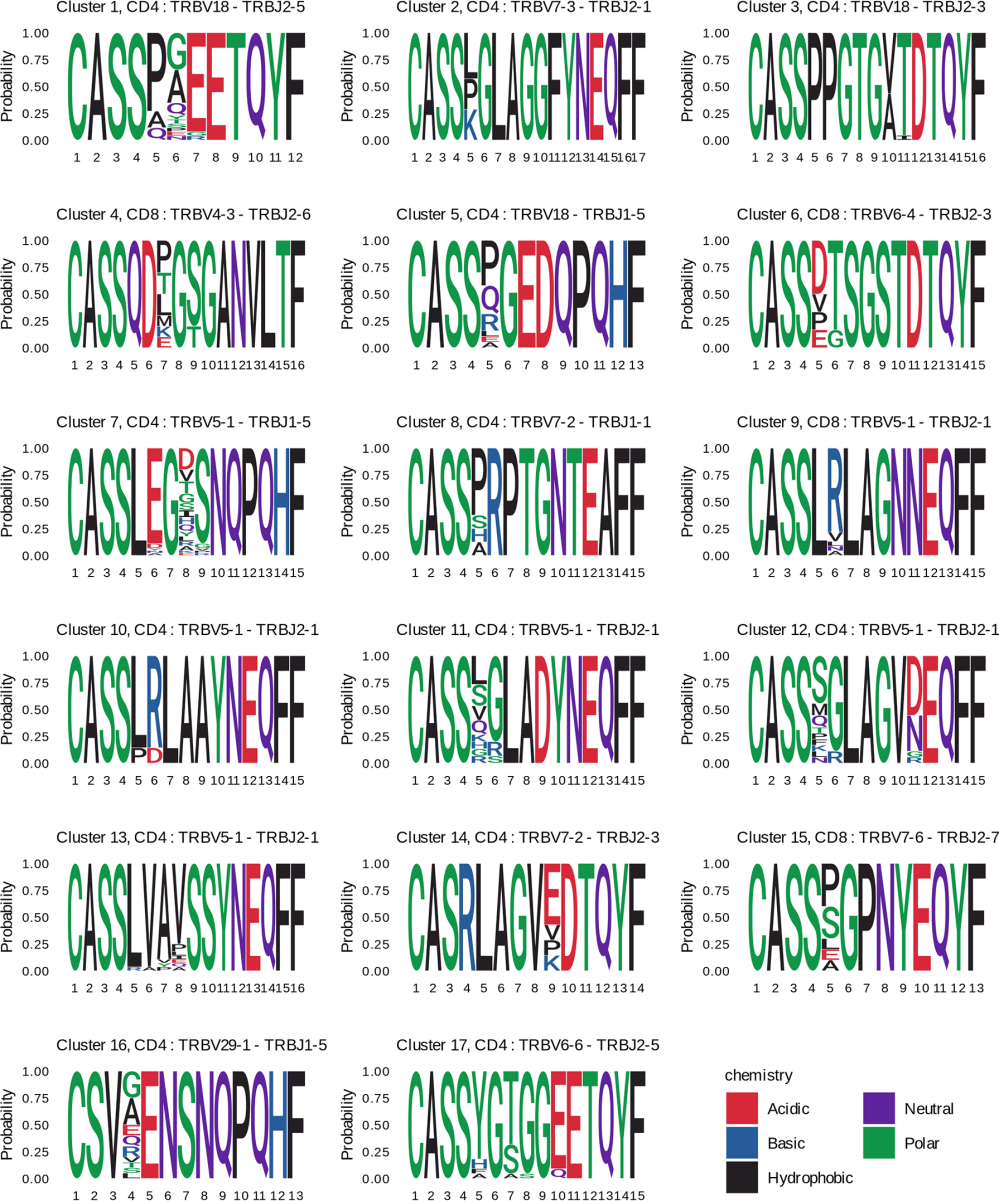
CDR3 amino acids motifs of clustered clonotypes from different donors. CDR3 amino acid sequences of the responded clonotypes from the indicated clusters are presented as sequence logos. Cluster numbers (as in **Figure 5**), phenotypes (CD4^+^ or CD8^+^), and V-J segments of T cell clones are shown on the top of the logos. Amino acids are colored according to their chemical properties.

The clonotypes of four clusters (4, 6, 9, and 15) were detected in TCRβ repertoires of the CD8^+^ T cell subset, and those of the other clusters – in the CD4^+^ T cell subset. Three and two clonotypes from clusters 8 and 17, respectively, were detected in the TCRβ repertoire of CD137^+^ T cells from donor #6 on day 44. For each cluster we identified CDR3 amino acid sequence motif (**Figure 6**), suggesting response to the same pMHC complex in donors with shared HLA alleles.

## 4 Discussion

Using high-throughput sequencing of the TCRβ repertoire, we revealed robust T cell clonal expansion in response to the licensed inactivated TBE vaccine (Tick-E-Vac^®^, FSBSI “Chumakov FSC R&D IBP RAS”). We confirmed vaccine-specificity of expanded clonotypes by *in vitro* stimulation with inactivated purified TBEV particles. The general dynamics of vaccine-associated clonotypes was more diverse than that of TBEV-specific IgGs, and the peak of expansion was observed on days 37 or 44 after the start of vaccination. These results indicate that the antibody response alone does not fully reflect individual differences in adaptive immune response to TBE vaccination and should be considered along with T cell characteristics. Furthermore, both time points (days 37 and 44 corresponding to one and two weeks after the 2nd dose, respectively) should be included in the analysis of T cell immune response to the TBE vaccine. Although both CD4^+^ and CD8^+^ T cell subpopulations were involved in immune response, the former clones were predominant. CD4^+^ vaccine-associated T cell clonotypes were found among IFNγ-producing T cells. Described CD4^+^ IFNγ-producing T cells belong to Th1 cells in most cases (32). Our results complement previous data on the functional properties of CD4^+^ T cell response to the inactivated TBE vaccine (29), but do not agree with the reported failure of this vaccine to induce a detectable CD8^+^ T cell response (33–36). Since CD8^+^ T cells are an essential component of protective immunity against viral infections, there is a need for new approaches to augment CD8^+^ T cell response to inactivated vaccines. One of such approaches could be the use of adjuvants that improve the efficiency of cross-presentation in dendritic cells (37).

We detected up to 79% of the responded clonotypes in the TCRβ repertoires of memory T cell subsets positive for CD45RO. This marker is expressed in various subsets of memory T cells, including T_CM_, T_TM_ and T_EM_ cells (38). We also observed enrichment of memory T cells with new vaccine-associated clonotypes after both the 1st and 2nd vaccine doses. About half (46 ± 17.7%) of the responded clonotypes detected in the memory subpopulation only after the 2nd dose were absent in the bulk repertoires on days -14, 0 and 30, suggesting that the 2nd immunization led to the recruitment of naive T cell clones along with reactivation of the existing memory T cells. Noteworthy, from 3% to 16.5% of the responded clonotypes were found in the memory T cell subset (but not in the bulk TCRβ repertoire) on day 0, i.e., before vaccination. These clonotypes had extremely low abundance and their proportion in all responded clonotypes did not correlate with the sequencing depth of the memory T cell repertoire (**Supplementary Figure 8**), suggesting that these preexisting memory T cell clones were reactivated because of TCR cross-specificity. In summary, our results demonstrate that the responded clonotypes produced a pool of memory T cells, which could be detected 1.5 months after the 2nd vaccine dose (day 75), and thus could provide long-term protection against TBEV infection.

The general dynamics of the TBE vaccine-associated clonotypes included several waves of clonal expansion. Using detailed clonal tracking, we identified four groups of the responded clonotypes with different expansion peaks, the most representative of which were those that peaked on days 37 or 44. In addition, these groups were abundant in the TCRβ repertoire of the memory T cell subset collected on day 30, i.e., before the 2nd dose. We suggest that clonotypes with peaks on days 37 and 44 were restimulated but had different proliferation rates, which could be due to several factors. Thus, the size of a T cell clone at the moment of stimulation, TCR affinity (39), cytokine background (40), and the functional state of T cells (41, 42) can all affect T cell clonal expansion. At the same time, some T cell clones from groups “d37” and “d44” could be recruited only after the 2nd vaccine administration.

Similar reasons for the “delayed” immune response may apply to the group with two peaks (days 30 and 44). The abundance on day 30 was measured at the contraction phase of immune response to the 1st vaccine dose, whereas that on day 44 was a result of expansion after the 2nd dose. However, instead of significant augmentation on day 44 (two weeks after restimulation) we observed comparable peaks for most clonotypes on days 30 and 44 (**Supplementary Figure 9**), which can indicate low proliferation capacity of the clonotypes.

In contrast to the groups described above, group “d30” did not show significant clonal expansion after the 2nd vaccine dose. One possible explanation of the weak response is an exhausted-like memory T cell phenotype of the clonotypes. Galletti et al. (43) have shown that exhausted-like memory T cell progenitors (T_PEX_) have lower proliferation rates and generate progeny with reduced functionality than progenitors of classic memory T cells. Authors identified T_PEX_ as CCR7^+^PD-1^+^TIGIT^+^(GZMK^+^) T cells, but also demonstrated that they can express CD45RO. Exhausted T cells reported in different types of infections could be generated after stimulation with a high antigen dose (44), which is the case for inactivated vaccines.

Convergent selection of antigen-specific clonotypes accompanies each T cell response; therefore, we expected to observe expansion of T cells with similar TCR sequences. Many clusters of similar vaccine-associated clonotypes were found in each donor, indicating recognition of diverse vaccine epitopes and generation of full-scale T cell-mediated defense. Several dominant clonotypes with high abundance at the expansion peak could be distinguished in many clusters, suggesting a higher affinity of their TCRs for the respective epitopes than that of the other clonotypes in the cluster. The differences in TCR affinity could lead to variations in clonotype dynamics within the same cluster.

Donors with shared HLA alleles responded to TBE vaccination with similar clonotypes, which formed 17 clusters. The majority of clonotypes were identified as CD4^+^, which can be due to preferential activation of CD4^+^ T cells in response to the inactivated TBE vaccine and to a greater diversity of CD4^+^ T cell clones, which provides a higher probability of TCRβ sequence matching. Earlier, Schwaiger et al. (45) have identified TBEV peptides representing the most promising candidate immunodominant epitopes for the activation of CD4^+^ T cells. Since donors in their and our study have matching HLA alleles, the data could be combined to engineer HLA class II multimers of TBEV epitopes for studying the immune response to this vaccine in detail.

At the same time, we observed several clusters of vaccine-associated CD8^+^ T cell clones in the TCRβ repertoires of different donors. For three clusters, we determined HLA class I alleles common to all the donors in the respective cluster: HLA-C*12:03 for cluster 4, HLA-C*07:02 and HLA-C*12:03 for cluster 9, and HLA-B*08:01 for cluster 15 (**Supplementary Table 2**). In previous studies, several immunodominant HLA-A2- and HLA-B7-restricted TBEV epitopes have been detected using T cells from TBEV-infected patients (46, 47). However, the identified peptides are derived from non-structural TBEV proteins, which usually are not present in inactivated TBE vaccines and, therefore, cannot be used for the analysis of immune response to vaccination. For this reason, HLA class I allele variants of donors with clustered CD8^+^ T cell clones should also be tested in the future, which would facilitate the search for universal TBEV epitopes to investigate immune response to both infection and vaccination.

In conclusion, we observed robust immune response to immunization with the inactivated TBE vaccine. Longitudinal tracking of clonal expansion enabled detection not only CD4^+^ but also CD8^+^ T cell response. The dynamics of vaccine-associated clonotypes was highly diverse, and all clonotype groups contributed to the generation of a memory T cell pool. The similarity of TCRs in the responded clonotypes indicated that the TBE vaccine presented a set of immunogenic peptides for both CD4^+^ and CD8^+^ T cells. Our findings should contribute to the development of more effective inactivated vaccines with a higher immunogenic potential.

## Data availability statement

The data presented in the study are deposited in the SRA repository, accession number PRJNA847436. The data can be found at https://www.ncbi.nlm.nih.gov/bioproject/PRJNA847436.

## Ethics statement

The studies involving human participants were reviewed and approved by Pirogov Russian National Research Medical University local ethics committee. The patients/participants provided their written informed consent to participate in this study.

## Author contributions

AS, IM, and YL contributed to conception and design of the study. AS, EkK, MP, AM, SU, MS, IZ, AK, and IM performed sample collection and preparation for sequencing at different stages. AS and EuK conducted antibody assays. AS, EkK, MS, IM, and YL performed data processing, statistical analysis and interpretation of results. AS, EkK, AM, MV, and YL wrote sections of the manuscript. All authors contributed to manuscript revision, read, and approved the submitted version.

## Funding

This work was supported by Russian Science Foundation grant (RSF 20-15-00351).

## Conflict of interest

The authors declare that the research was conducted in the absence of any commercial or financial relationships that could be construed as a potential conflict of interest.

## Publisher’s note

All claims expressed in this article are solely those of the authors and do not necessarily represent those of their affiliated organizations, or those of the publisher, the editors and the reviewers. Any product that may be evaluated in this article, or claim that may be made by its manufacturer, is not guaranteed or endorsed by the publisher.
