# Supplementary material for: Inactivated tick-borne encephalitis vaccine elicits several overlapping waves of T cell response
Source: Front Immunol. 2022 Aug 24;13:970285. doi: 10.3389/fimmu.2022.970285 (PMC9449805; doi:10.3389/fimmu.2022.970285)
Supplement: Supplementary file 1 [file DataSheet_1.zip › Sycheva_et_all_suppl_material_revised.docx]

Supplementary Material

**Supplementary Figure 1.** Gating strategy for isolation of CD3^+^IFNγ^+^ (A) and CD3^+^CD137^+^ (B) cells after *in vitro* stimulation.

**Supplementary Figure 2.** TCRβ clonotype frequency scatterplots (log_10_ scale) for pairs of replicates or time points (example for donor #5). **(A, B)** Clonotype frequency in the bulk TCRβ repertoires of two replicates on day 0 (A) and day 37 (B). **(C)** Clonotype frequency in the bulk TCRβ repertoires on day 0 vs. day 37. Frequency in the bulk TCRβ repertoire at time point was calculated as the average clonotype frequencies in two replicates of the corresponding time point. Clonotypes with significantly increased frequency (32-fold threshold) are colored red.

**Supplementary Figure 3.** Dynamics of the response to TBE vaccination. **(A)** Fraction of vaccine-associated clonotypes in the bulk TCRβ repertoire calculated as the sum of the average clonotype frequencies in two replicates at each time point. **(B)** Level of TBEV-specific IgGs.

**Supplementary Figure 4.** Dynamics of responded clonotypes. Fractions of bulk (solid line), CD4^+^ (dashed line) and CD8^+^ (dash-dotted line) responded T cell clones in the bulk TCRβ repertoire calculated as the sum of the average clonotype frequencies in two replicates at each time point.

**Supplementary Figure 5.** Vaccine-associated and in vitro activated T cell clones. Vaccine-associated clonotypes found in one (“single”) or two (“double”) TCRβ repertoires of CD137^+^ and IFNγ-producing T cells collected on days 37 and 44 from donors #5 (A) and #6 (B). For “double” clonotypes, each column corresponds to one clonotype; for “single” clonotypes, the numbers indicate clonotypes detected in the CD137^+^ and IFNγ-producing T cell repertoires.

**Supplementary Figure 6.** Sequence similarity networks for the responded clonotypes from all donors. Each vertex corresponds to a unique TCRβ clonotype; only clusters with 4 or more clonotypes are shown. The vertex size corresponds to the maximum abundance of a clonotype (at the peak of expansion); filled vertices indicate clonotypes identified with the ‘edgeR’ package. Edges connect clonotypes with one or no mismatches in CDR3 amino acid sequences and identical V and J segments. Only edges between vertices corresponding to clonotypes of the same donor are displayed. Donor #3 with 4249 clonotypes in clusters is not shown.

**Supplementary Figure 7.** Heatmap with number of HLA alleles shared between two donors. For a full list of HLA alleles see Supplementary Table 2.

**Supplementary Figure 8.** Correlation between the proportion of vaccine-associated clonotypes identified in the TCRβ repertoire of memory T cells on days 0 (blue), 30 (yellow), and 75 (gray), and the total number of clonotypes in the repertoire of the respective memory T cell subset. Two-tailed Pearson correlation coefficient (95% CI) was used to assess the significance of correlation.

**Supplementary Figure 9.** Dynamics of the group “d30_44” fraction in the bulk TCRβ repertoire. The abundance of each clonotype is shown in different colors.
