# Supplementary figures and images for: Inactivated tick-borne encephalitis vaccine elicits several overlapping waves of T cell response
Source: Front Immunol. 2022 Aug 24;13:970285. doi: 10.3389/fimmu.2022.970285 (PMC9449805; doi:10.3389/fimmu.2022.970285)

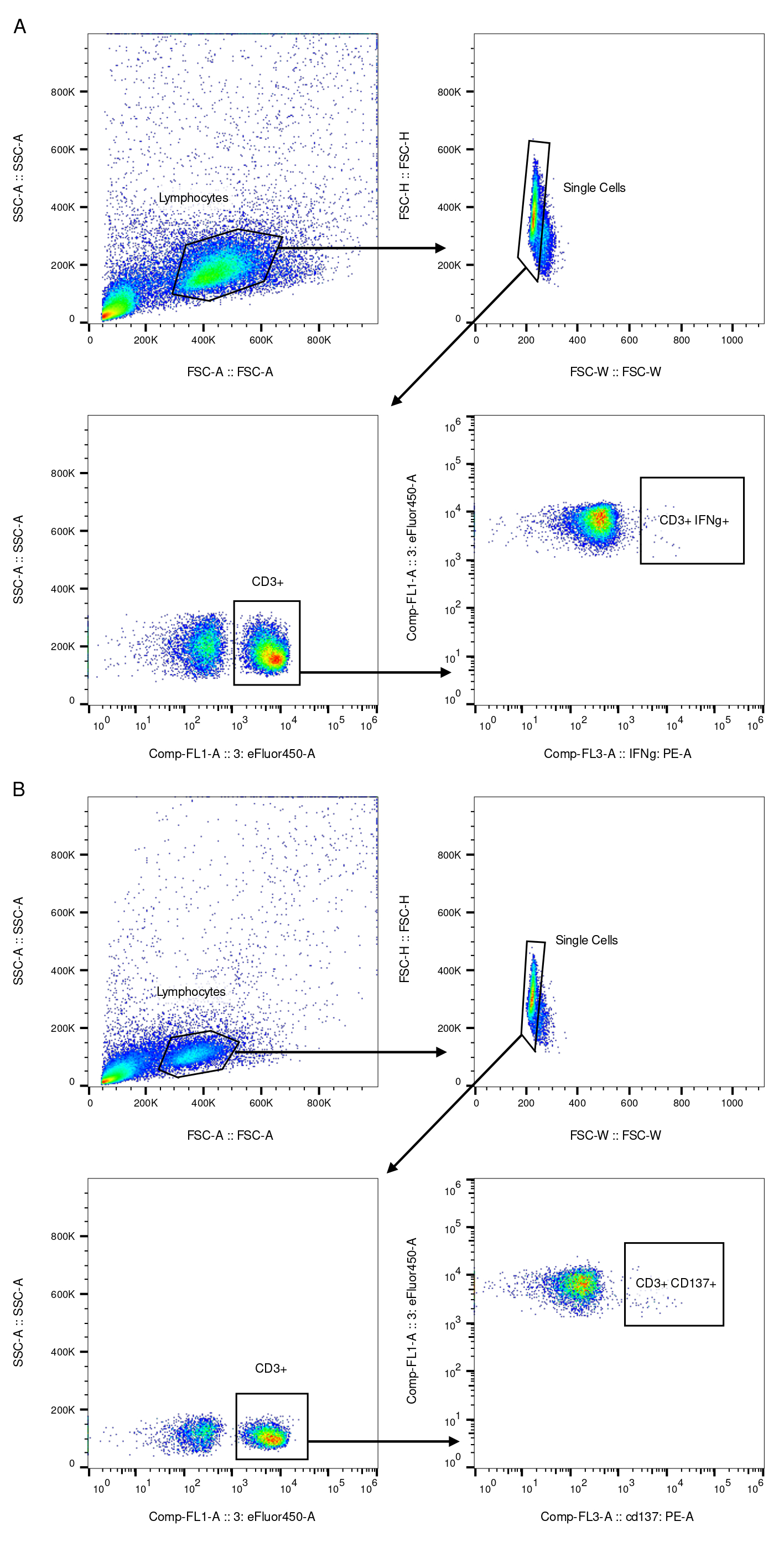

Supplement: Supplementary file 1 [file DataSheet_1.zip › Suppl_Fig_1.jpg]

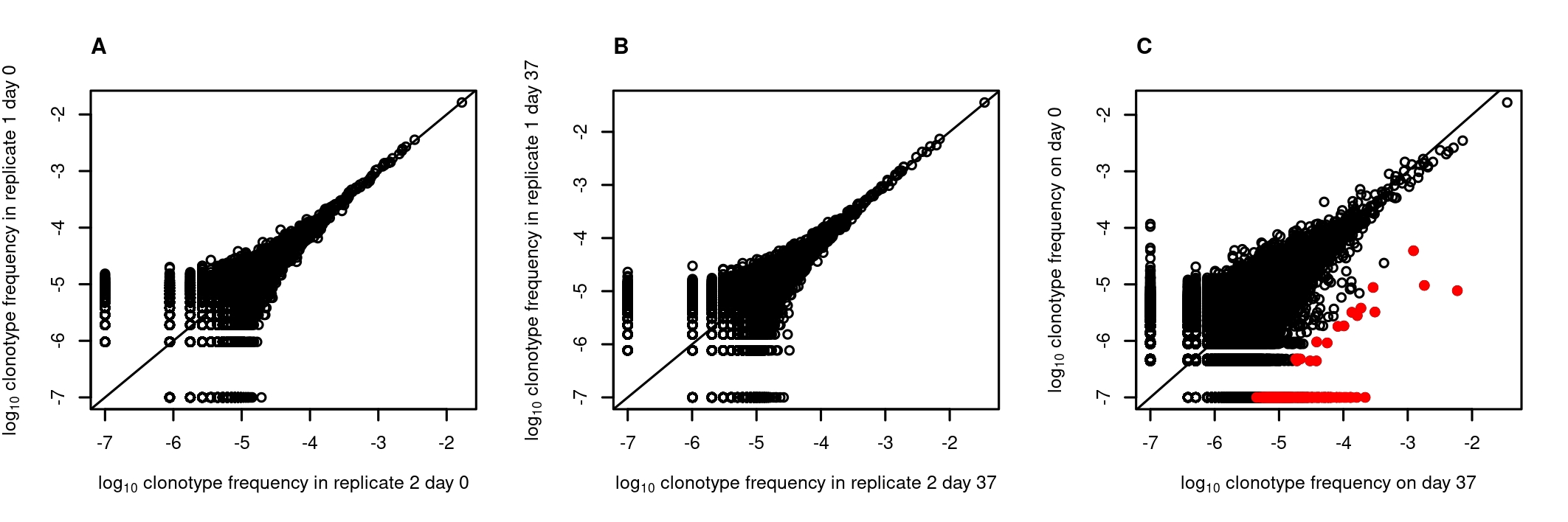

Supplement: Supplementary file 1 [file DataSheet_1.zip › Suppl_Fig_2.jpg]

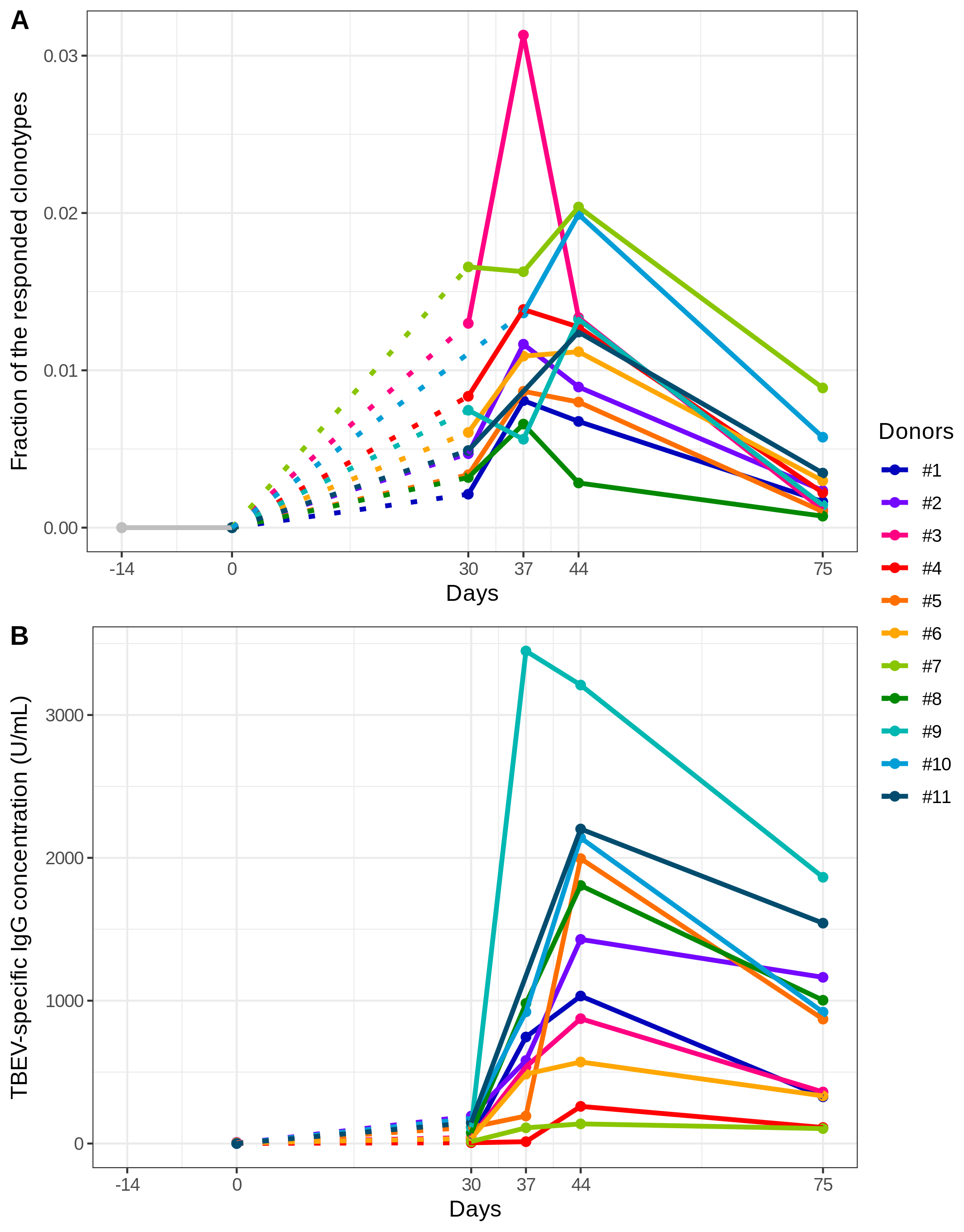

Supplement: Supplementary file 1 [file DataSheet_1.zip › Suppl_Fig_3.jpg]

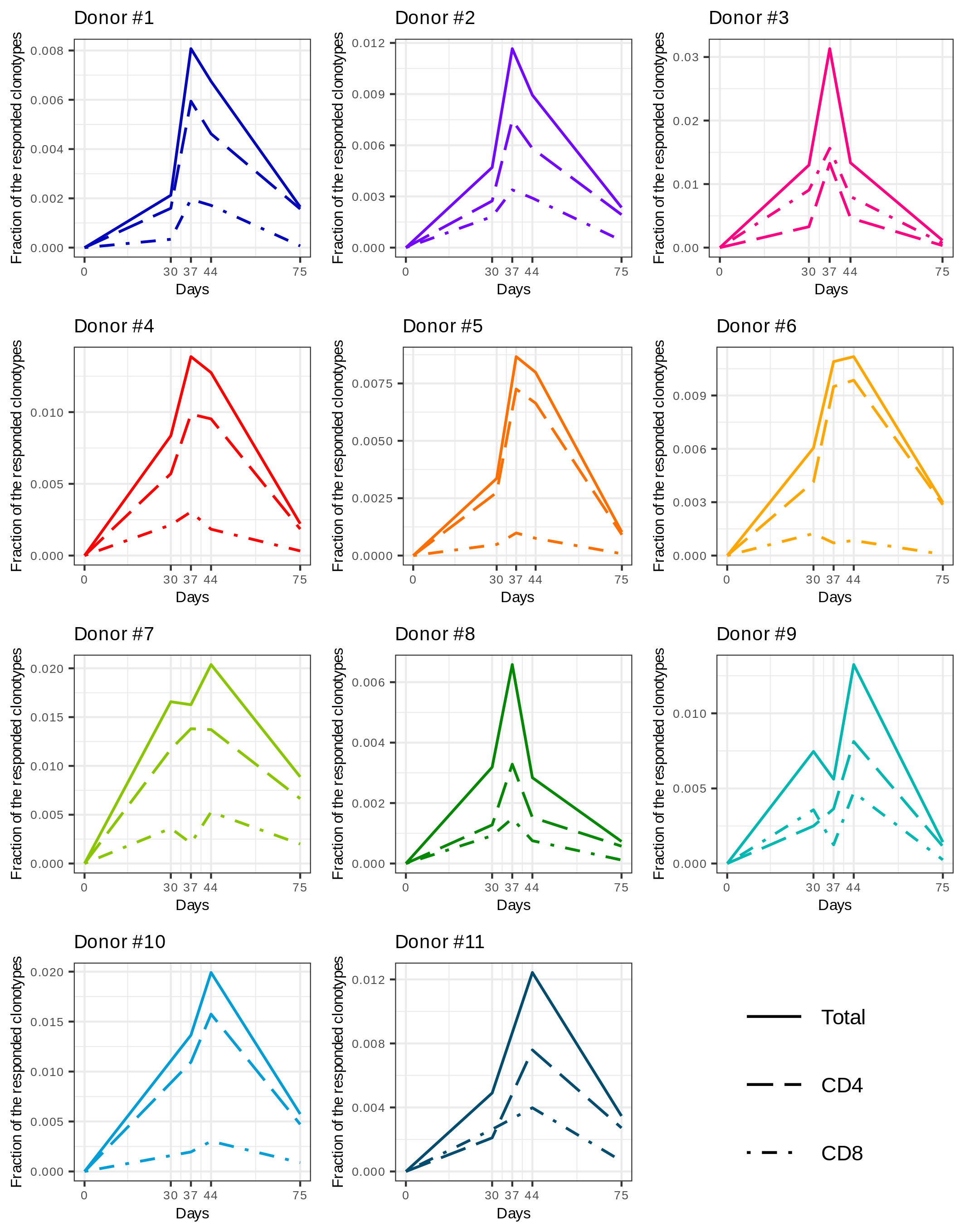

Supplement: Supplementary file 1 [file DataSheet_1.zip › Suppl_Fig_4.jpg]

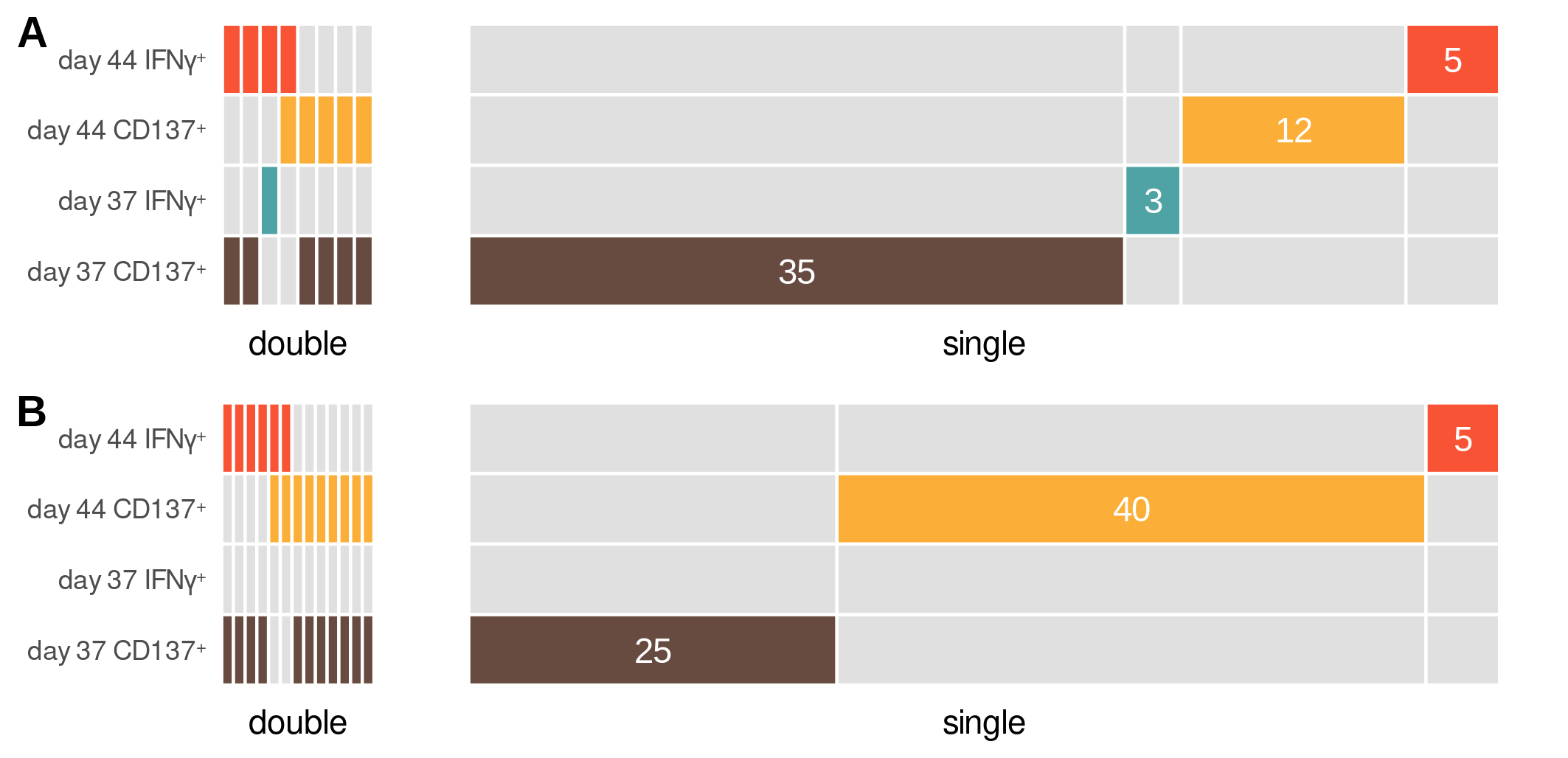

Supplement: Supplementary file 1 [file DataSheet_1.zip › Suppl_Fig_5.jpg]

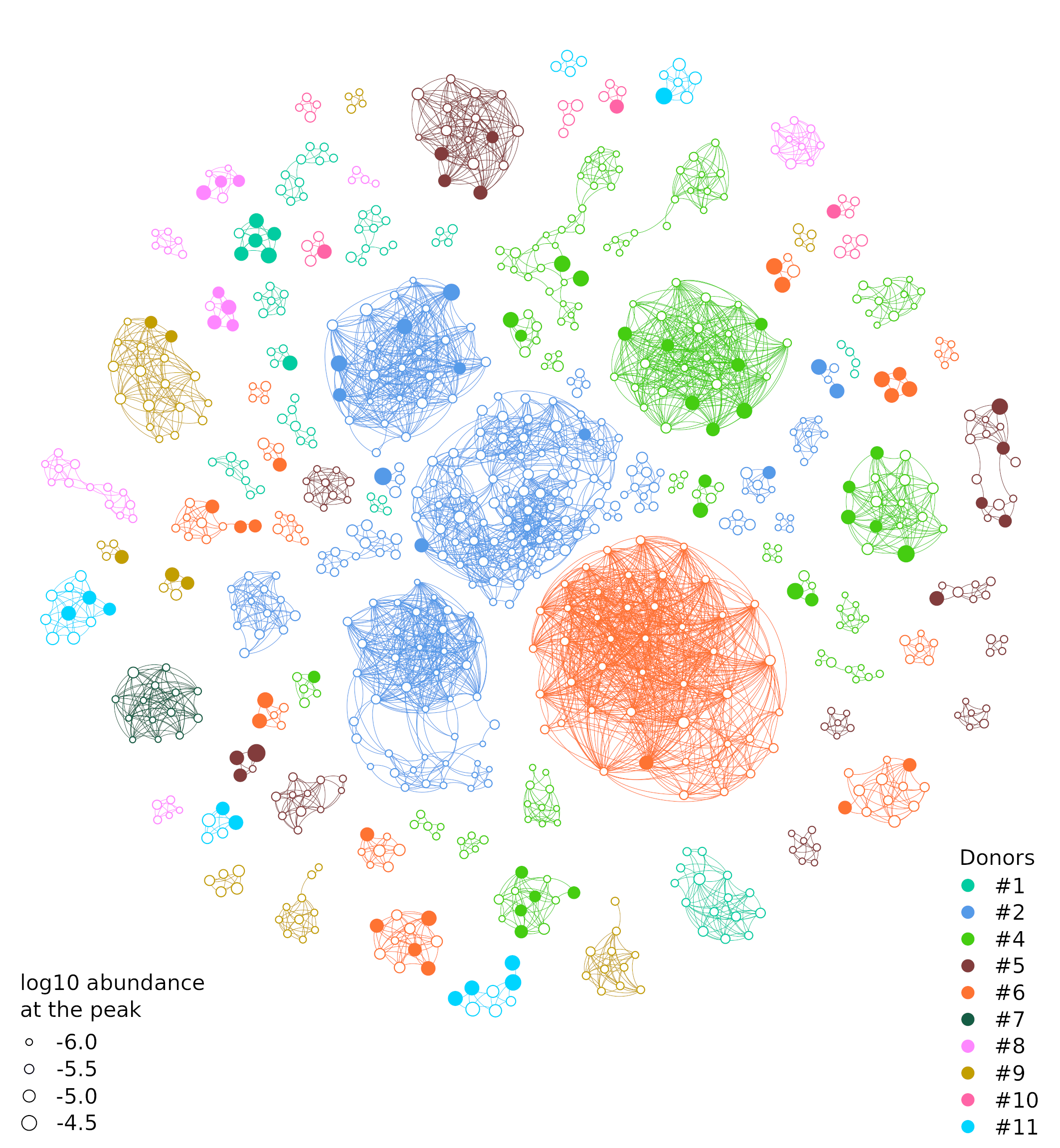

Supplement: Supplementary file 1 [file DataSheet_1.zip › Suppl_Fig_6.jpg]

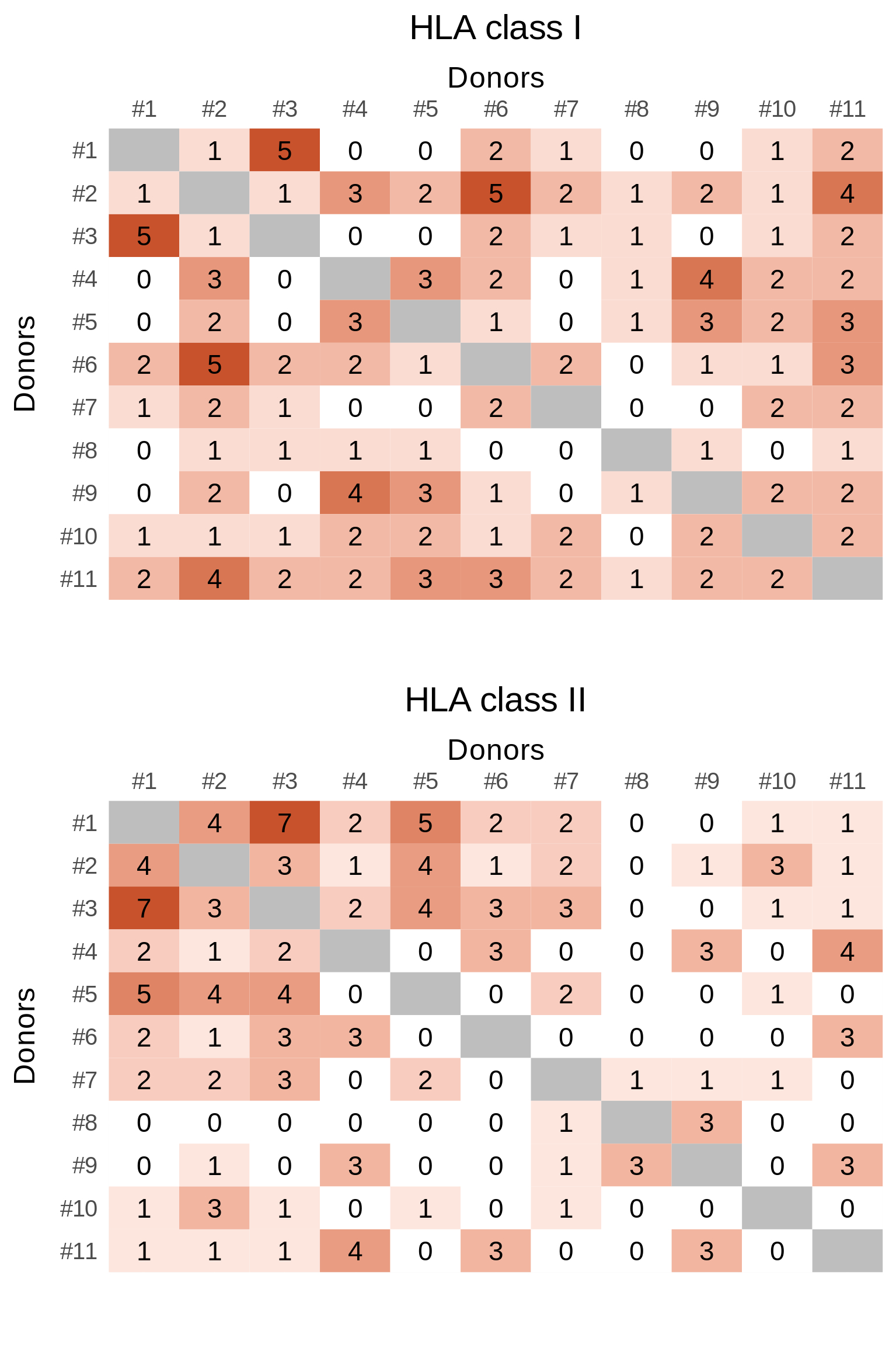

Supplement: Supplementary file 1 [file DataSheet_1.zip › Suppl_Fig_7.jpg]

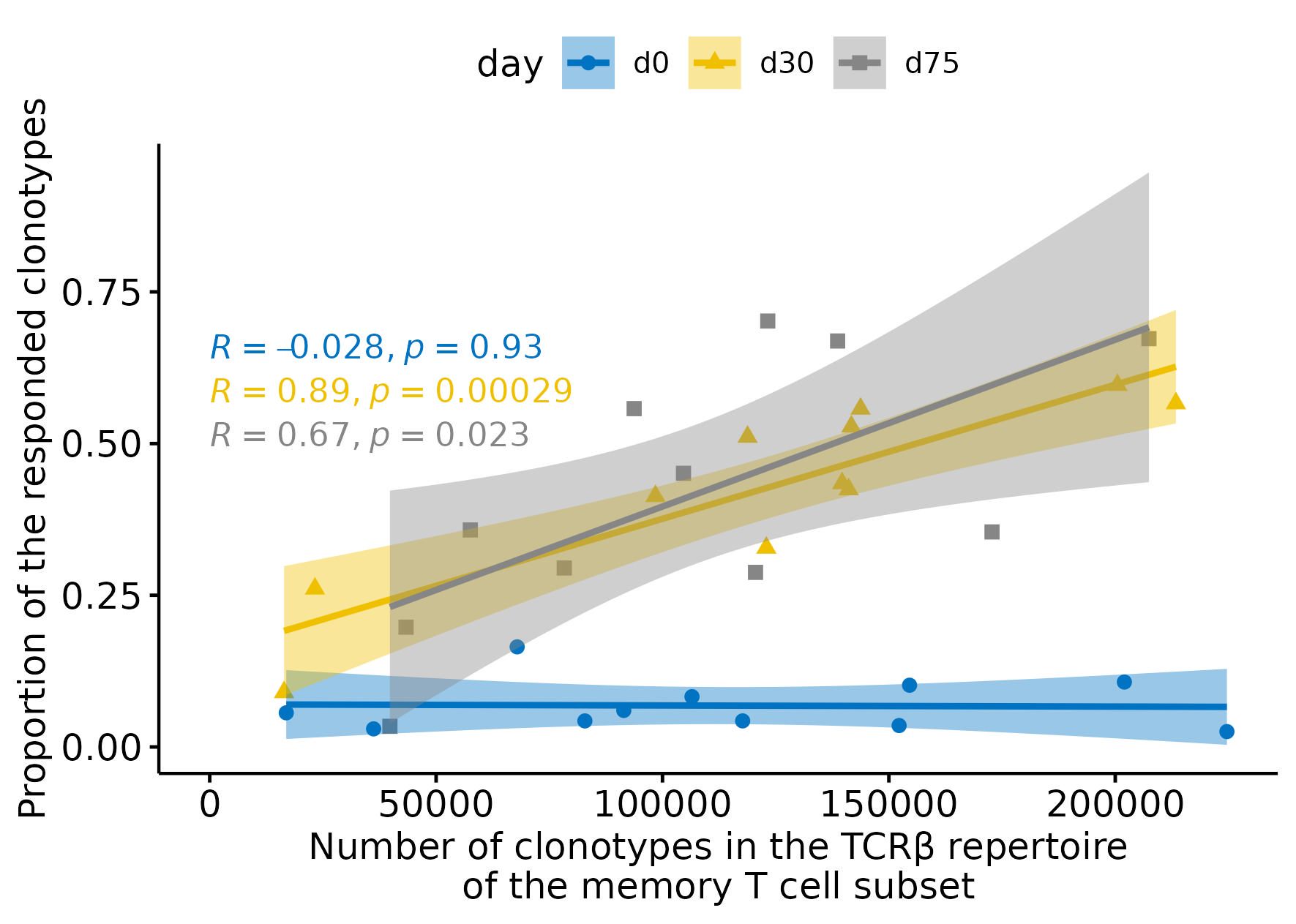

Supplement: Supplementary file 1 [file DataSheet_1.zip › Suppl_Fig_8.jpg]

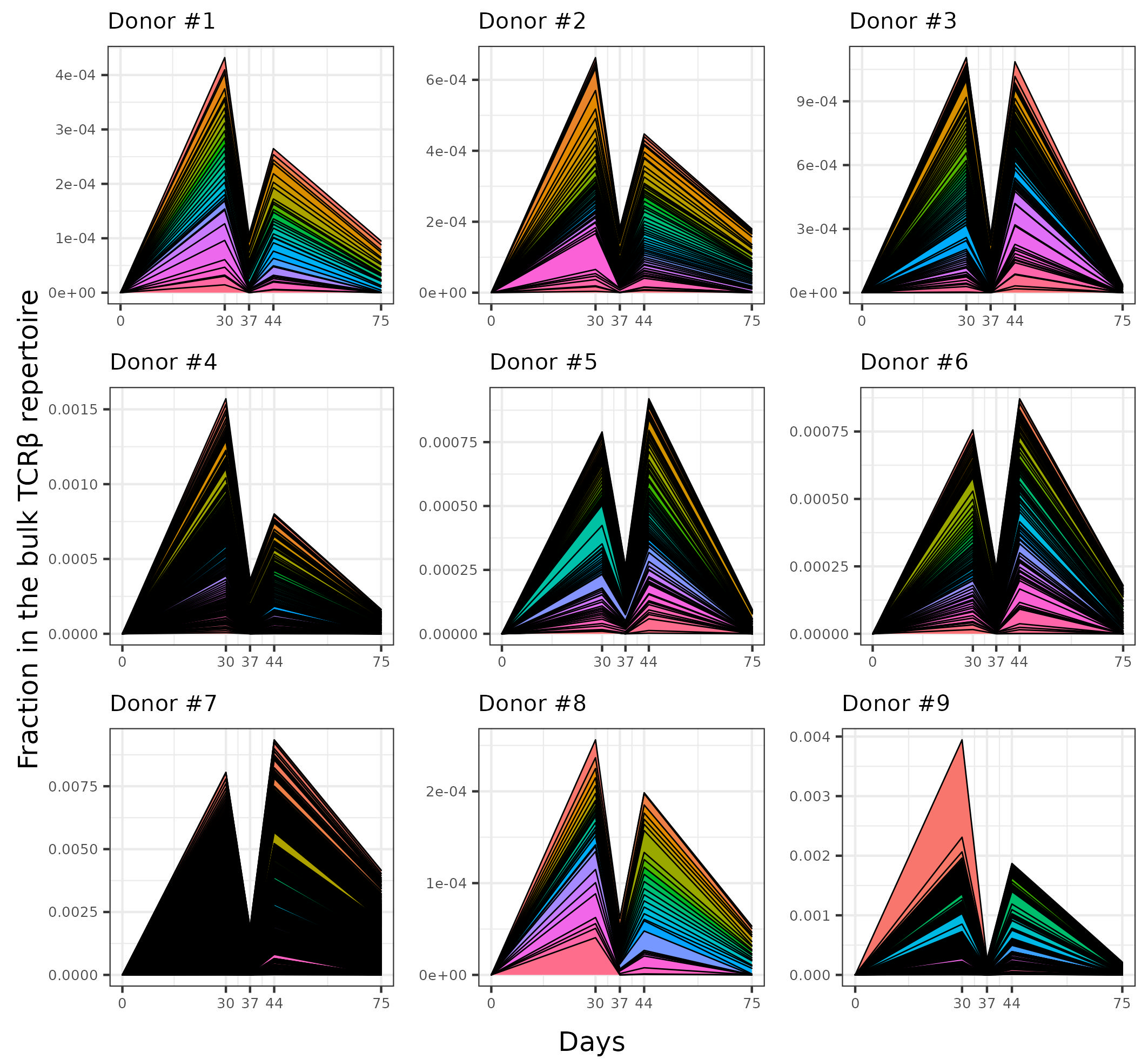

Supplement: Supplementary file 1 [file DataSheet_1.zip › Suppl_Fig_9.jpg]
